# Imaging Glioblastoma Response to Radiotherapy Using ^2^H Magnetic Resonance Spectroscopy Measurements of Fumarate Metabolism

**DOI:** 10.1158/0008-5472.CAN-22-0101

**Published:** 2022-08-16

**Authors:** Friederike Hesse, Alan J. Wright, Vencel Somai, Flaviu Bulat, Felix Kreis, Kevin M. Brindle

**Affiliations:** 1 Cancer Research UK Cambridge Institute, University of Cambridge, Cambridge, United Kingdom.; 2 Department of Radiology, School of Clinical Medicine, Cambridge Biomedical Campus, University of Cambridge, Cambridge, United Kingdom.; 3 Department of Chemistry, University of Cambridge, Cambridge, United Kingdom.; 4 Department of Biochemistry, University of Cambridge, Cambridge, United Kingdom.

## Abstract

Early detection of tumor cell death in glioblastoma following treatment with chemoradiation has the potential to distinguish between true disease progression and pseudoprogression. Tumor cell death can be detected noninvasively *in vivo* by imaging the production of [2,3-^2^H_2_]malate from [2,3-^2^H_2_]fumarate using ^2^H magnetic resonance (MR) spectroscopic imaging. We show here that ^2^H MR spectroscopy and spectroscopic imaging measurements of [2,3-^2^H_2_]fumarate metabolism can detect tumor cell death in orthotopically implanted glioblastoma models within 48 hours following the completion of chemoradiation. Following the injection of [2,3-^2^H_2_]fumarate into tumor-bearing mice, production of [2,3-^2^H_2_]malate was measured in a human cell line–derived model and in radiosensitive and radioresistant patient-derived models of glioblastoma that were treated with temozolomide followed by targeted fractionated irradiation. The increase in the [2,3-^2^H_2_]malate/[2,3-^2^H_2_]fumarate signal ratio posttreatment, which correlated with histologic assessment of cell death, was a more sensitive indicator of treatment response than diffusion-weighted and contrast agent–enhanced ^1^H MRI measurements, which have been used clinically to detect responses of glioblastoma to chemoradiation. Overall, early detection of glioblastoma cell death using ^2^H MRI of malate production from fumarate could help improve the clinical evaluation of response to chemoradiation.

**Significance:**

^2^H magnetic resonance imaging of labeled fumarate metabolism can detect early evidence of tumor cell death following chemoradiation, meeting a clinical need to reliably detect treatment response in glioblastoma.

## Graphical Abstract

**Figure:**
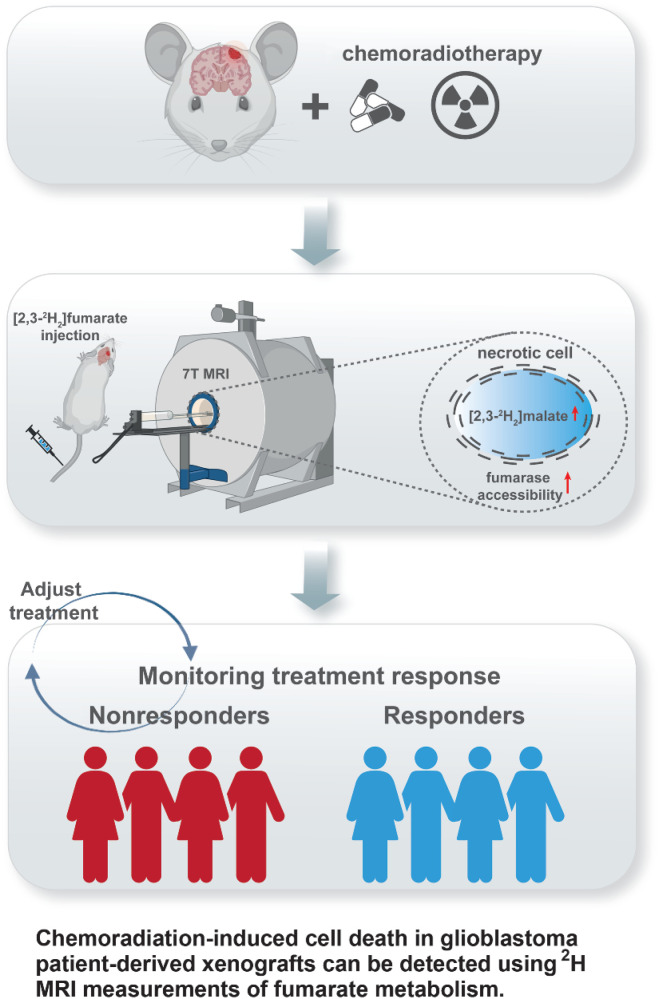

## Introduction

Glioblastoma is the most common of all malignant brain tumors (1, 2) and remains the deadliest primary malignant brain tumor, with a median survival of 15 months (3). Standard-of-care treatment comprises debulking surgical resection followed by treatment with temozolomide and fractionated targeted radiation (3, 4).

The response of glioblastoma to radiotherapy is typically monitored using contrast agent–enhanced MRI, where response is detected through a decrease in the size of the enhancing lesion using the response assessment in neuro-oncology criteria (5–8). However, the technique does not measure the activity of the tumor cells but the integrity of the blood-brain barrier (BBB), which when opened leads to increased contrast agent enhancement. Consequently it can be difficult to distinguish disease progression from pseudoprogression, which occurs in approximately 30% of patients and is defined as a new or enlarging area or areas of contrast agent enhancement that occur early after the end of treatment but in the absence of true tumor growth (9). Such increases may result from a transient increase in permeability of the tumor vasculature after irradiation (9), or radio-necrosis, which can also result in a disrupted BBB and oedema and usually occurs 6 to 12 months after radiotherapy (10, 11). Thus, changes in BBB permeability can influence the degree and extent of the enhancing area irrespective of the size and activity of the tumor (5, 12). Therefore, methods that assess the activity of the tumor more directly are expected to provide a more reliable distinction between true disease progression and pseudoprogression. It is notable that of the advanced imaging techniques that have been developed to distinguish between these two scenarios magnetic resonance spectroscopy (MRS) measurements of tumor cell metabolism have shown the best sensitivity and specificity (13). An alternative to measuring the metabolic activity of the tumor cells posttreatment is to measure tumor cell death directly, which has the potential to indicate longer-term outcomes (14).

We have shown previously that tumor cell death posttreatment can be detected *in vivo* by imaging the metabolism of hyperpolarized [1,4-^13^C_2_]fumarate using ^13^C magnetic resonance spectroscopic imaging (MRSI; refs. 15–17). Fumarate, which is hydrated in the reaction catalyzed by fumarase to produce malate, is taken up slowly by viable cells. When a cell becomes necrotic the injected hyperpolarized [1,4-^13^C_2_]fumarate rapidly gains access to the enzyme via the permeabilized plasma membrane, either intracellularly or extracellularly, and there is an increase in the rate of labeled malate production. Fumarase requires no coenzyme, which could be depleted by cell necrosis, needing only water and fumarate to produce malate. ^13^C MRSI of hyperpolarized [1,4-^13^C_2_]fumarate metabolism has also been used to detect cell death in models of myocardial infarction (18) and acute kidney necrosis (19). A limitation of imaging with hyperpolarized ^13^C-labelled substrates is the short polarization lifetime (2–3 minutes *in vivo*), which in the case of hyperpolarized [1,4-^13^C_2_]fumarate limits the build-up of detectable malate. We have shown recently that by using deuterated [2,3-^2^H_2_]fumarate and monitoring its conversion to [2,3-^2^H_2_]malate over a much longer time period using ^2^H MRS and MRSI measurements that we can detect a much larger increase in the [2,3-^2^H_2_]malate/[2,3-^2^H_2_]fumarate signal ratio in necrotic cells (20). This makes it a potentially more sensitive method for detecting cell death than imaging with hyperpolarized [1,4-^13^C_2_]fumarate. We show here that ^2^H MRSI measurement of [2,3-^2^H_2_]fumarate metabolism is a more sensitive method for detecting tumor cell death in patient-derived orthotopically implanted xenograft (PDX) models of glioblastoma following chemoradiation than ^13^C MRSI measurements with hyperpolarized [1,4-^13^C_2_]fumarate or contrast agent enhanced or diffusion-weighted ^1^H MRI measurements.

## Materials and Methods

### Cell culture

Patient-derived cell lines, A11 (passage 24), S2 (passage 11), were obtained from Prof. Colin Watts. Cells were grown as monolayer cultures on extracellular matrix–coated flasks in phenol red–free Neurobasal A medium (Gibco, UK) containing 2 mmol/L l-glutamine (Sigma, UK), 1% streptomycin/penicillin (Invitrogen, UK) 20 ng/mL hEGF (Sigma, UK), 20 ng/mL hFGF (R&D Systems, UK), 2% B27 (Invitrogen, UK), and 1% N2 (Invitrogen, UK). When confluent the cells were washed with Hank's Balanced Salt Solution (Gibco, UK) and dissociated using Accutase (Sigma, UK). The human glioblastoma cell line, U87 (passage 51; ATCC, catalog no. HTB-14, RRID:CVCL_0022), was cultured in DMEM supplemented with 10% FBS (Gibco, UK). When confluent, cells were washed with PBS and harvested using 0.25% trypsin (Gibco, UK). A11, S2, and U87 cells were implanted after two passages from thawing. Viability and cell number were assessed using a Vi-Cell counter (Vi-Cell XR, Beckman Coulter, RRID:SCR_019664). Cells tested negative for *Mycoplasma*, based on the Phoenix qPCR Mycoplasma kit and were STR genotyped on February 25, 2022, using the PowerPlex_16HSM_Cell Line panel and analyzed in-house using Applied Biosystems GeneMapper 5 software. It yielded a 100% match to cells in the Cellosaurus STRdatabase (U87) or to the in-house reference profile when it was established (S2, A11).

### Orthotopic tumor implantation

Procedures were performed in compliance with project and personal licenses issued under the United Kingdom Animals Scientific Procedures Act, 1986 and were approved by an Animal Welfare and Ethical Review Body. Twelve-week-old (20 g) female BALB/c nude mice (Charles River Laboratories, RRID:SCR_003792) were anaesthetized by inhalation of 1% to 2% isoflurane in air/O_2_ (75%/25%, 2 L/min). Analgesia [0.3 mg/mL buprenorphine hydrochloride and 0.135% w/v chlorocresol diluted 1:10 in 0.9% sodium chloride and 1 mL/kg of subcutaneous Rimadyl LA (Pfizer) containing carpofen (Zoetis; 5 mg/kg diluted in 1:10 in 0.9% sodium chloride] was administered subcutaneously. Animals were placed in a stereotactic surgical frame (Kopf) and the head fixed using ear and bite bars. A 1-mm burr hole was drilled 2-mm anterior and 3-mm lateral to the bregma and a syringe containing 5 μL of 0.5 × 10^6^ cells/μL inserted 3.5 mm intracranially. Upon withdrawal by 0.5 mm, the contents of the syringe were injected into the right frontal lobe at 2 μL/min. The burr hole was filled with bone wax and sutures used to close the wound, which was covered using tissue glue (GLUture). Metacam (10 mg/kg) was provided 48 hours and 72 hours postsurgery as analgesia. Animals were assigned randomly to experimental groups. Three animals developed hydrocephalus ∼3 weeks after intracranial implantation and were excluded from the study.

### Image-guided cranial irradiation

Mice were anaesthetized with isoflurane and placed on an irradiator bed (SARRP, XStrahl Inc.). Treatment planning and radiation delivery were performed using MuriPlan software (XStrahl Inc.). CT images were acquired at 60 kV and 0.8 mA, with a voxel size of 0.275 mm, which delivers a dose of 1.2 cGy (21). Tissue segmentation was adjusted for each animal to define bone, tissue, and air. Field isocenters were applied to the segmented images to target radiotherapy delivery, and a collimator was used to customize the radiotherapy field. The SARRP delivers 225-kVp X-rays at 13-mA current (22), through a 0.15-mm Cu filter, at a dose rate of 3.1 Gy/min, and allows exact anatomic targeting with delivery of beams down to 0.5 mm. Mice received a 90° brain arc field (–45° to 45°), with a field size of 5×5 mm^2^. Animals were treated with 5 Gy per day for 4 days. Temozolomide (100 mg/kg) was administered by oral gavage 1 hour prior to irradiation. Treatment planning and delivery simulates the clinical situation (23). Mice were euthanized by cervical dislocation when they developed symptoms.

### ^2^H MR spectroscopy measurements on media samples

A11, S2, and U87 cells, growing in culture medium, were treated with 50 μmol/L temozolomide 1 hour prior to each radiotherapy session (5 Gy per day, 15 Gy total, delivered over 110 seconds using a Cs-137 irradiator (IBL 637; CIS Bio International).

At 24 hour following the last treatment, the cells were washed in PBS, resuspended at 1×10^6^ cells/mL in 10 mL culture medium, and 5 mmol/L [2,3-^2^H_2_]fumarate was added. One milliliter samples were taken at the specified time points, centrifuged at 1,000 *g*, and the supernatants transferred to 5-mm tubes. A separate flask was used for each time point. Formate-d (5 mmol/L) was added to the samples as a reference and ^2^H nuclear magnetic resonance (NMR) spectra were acquired at 310 K using the ^2^H coil of a 5-mm ^1^H/broadband inverse detection probe in a 14.1 T NMR spectrometer (Bruker Spectrospin Ltd.) with a 90° pulse, a repetition time (TR) of 3 seconds with a 2,000 Hz spectral width into 1,024 data points and were the sum of 1,024 transients. Spectra were phased, baseline corrected, and peak integrals calculated using Topspin (Bruker Spectrospin Ltd., RRID:SCR_014227). The amplitudes of the water, fumarate, and malate resonances were normalized to the formate-d peak, after correction for formate-d resonance saturation. Absolute concentrations were obtained by correcting for the numbers of deuterons per molecule. The rate of deuterated malate production and the rates of water labeling were determined by linear least squares fitting of the concentrations using a MATLAB script (MATLAB, RRID:SCR_001622).

### ^2^H MR spectroscopy and spectroscopic imaging *in vivo*

Tumors obtained by orthotopic implantation of A11, S2, and U87 cells were treated when they were >60 mm^3^ (at ∼3.5 weeks for U87, ∼3.5 months for A11, and ∼7 months for S2 tumors). Animals were anesthetized by inhalation of 2% isoflurane in air/O_2_ (75%/25%, 2 L/min). Breathing rate and body temperature were monitored and body temperature maintained with a stream of warm air. ^2^H imaging and spectroscopy were performed at 7 T (Agilent, Palo Alto, CA); ^2^H resonance frequency 46.007MHz. Tumors were localized in axial ^1^H images acquired using a T_2_-weighted a fast spin echo (FSE) pulse sequence at 7 and 9.4 T (7T: TR, 2 s; TE, 50 ms; field-of-view (FOV), 32×32 mm^2^, 256×256 matrix; slice thickness, 1 mm; 10 slices. 9.4 T: TR, 2.2 s; TE, 50 ms; FOV, 35×35 mm^2^; 256×256 matrix; slice thickness, 1 mm; 10 slices). Disodium [2,3-^2^H_2_]fumarate was dissolved in water at a concentration of 312.5 mmol/L and ∼0.2 mL infused via a tail vein catheter. Infusion started 5 minutes after the start of spectral or image acquisition to give 1 g/kg body weight [2,3-^2^H_2_] disodium fumarate infused over a period of 20 minutes. Serial ^2^H spectra were acquired with a 2-ms BIR4 pulse (24), with a nominal flip angle of 67°, and a TR of 140 ms (20). Spectra were zero- and first-order phase corrected, and the peaks fitted with the AMARES toolbox (25, 26). Localization of signal to the tumors was achieved by the excitation profile of the surface coil and confirmed by imaging. 3D chemical shift images (CSI) were acquired with a 2-ms BIR4 pulse, with a nominal flip angle of 50°, with phase-encoding gradients encoding a 9×9×3 k-space matrix with a FOV of 27×27×27 mm^3^. Data were acquired into 256 complex points with a sweep width of 4 kHz and a TR of 140 ms (27).

### Hyperpolarization of [1,4-^13^C_2_, 2,3-^2^H_2_]fumarate

A 40-mg sample of [1,4-^13^C_2_, 2,3-^2^H_2_] fumaric acid (3.4 mmol/L, Cambridge Isotope Laboratories) dissolved in 8.74 mmol/L of DMSO containing 12 mmol/L of a trityl radical [(Tris(8-carboxy-2,2,6,6(tetra(methoxyethyl) benzo- [1,2–4,5′]bis-(1,3)dithiole-4-yl)methyl sodium salt; AH111501; GE Healthcare] and 0.8 mmol/L of an aqueous solution of a gadolinium chelate (Dotarem, Guerbet) was hyperpolarized in a 3.35-T Hypersense polarizer (Oxford Instruments). The frozen sample was dissolved in 6-mL phosphate buffer containing 40-mmol/L phosphate, 50-mmol/L NaCl, 40-mmol/L NaOH, 100-mg/L EDTA, heated to 180°C and pressurized to 10 bar to yield a final fumarate concentration of 20 mmol/L. The polarization ranged from 7% to 10%. Deuterated ^13^C-labelled fumarate was used with the anticipation that this might extend the polarization lifetime. However, the ^13^C T_1_ in PBS at 7T was 23.2 ± 2.5 s (*n* = 3; Supplementary Fig. S1) and therefore showed no evidence that this would significantly extend polarization lifetime at this magnetic field. Deuterium isotope effects in the reaction catalyzed by fumarase are small (28).

### ^13^C MR spectroscopy and spectroscopic imaging *in vivo*

Animals were anaesthetized as described above, a catheter inserted into the tail vein and the animal placed in a 7.0 T horizontal bore magnet (Agilent) with an actively decoupled dual-tuned ^13^C/^1^H volume transmit coil (Rapid Biomedical) and a 20-mm diameter ^13^C receive coil (Rapid Biomedical). Axial ^1^H images were acquired using a FSE acquisition sequence (30° pulse; TR, 2,000 ms; TE, 12 ms; FOV, 32 × 32 mm^2^, 256 × 256 matrix with 12 averages; slice thickness, 1 mm; 8 slices). The tumor slice was selected from these images and a slice selective excitation pulse and acquire sequence was used for ^13^C spectroscopy. After injection of 0.2 mL hyperpolarized 20 mmol/L [1,4-^13^C_2_, 2,3-^2^H_2_]fumarate, 60 ^13^C spectra were acquired from an 8-mm thick tumor slice at 3-second intervals with a flip angle of 25°.

### MR spectroscopy of tissue extracts

A11 and S2 tumor-bearing animals were injected with 1 g/kg [2,3-^2^H_2_]fumarate (*n* = 6 for each model, 3 treated and 3 untreated) under isoflurane anesthesia and after 20 minutes the animals were killed by cervical dislocation, the brains removed and cut in half transversely. The frontal lobe containing the tumor and the other half of the brain were freeze-clamped in liquid nitrogen-cooled tongs. Frozen samples were homogenized in ice-cold 2-mol/L PCA using a Precellys Cryolys Evolution tissue homogenizer (Bertin Instruments) and neutralized with 2-mol/L KOH. After centrifugation for 15 minutes at 13,000 *g*, 200 μL of the supernatant was mixed with 300 μL of H_2_O and a formate-d standard added to a final concentration of 4 mmol/L. ^2^H NMR spectra were acquired using the same acquisition parameters as used for the media samples. Concentrations were calculated by comparison of the signal intensities with that of the formate-d standard. Following the ^2^H NMR measurements, a standard for proton NMR measurements, 3-(trimethylsilyl)-2,2,3,3-tetradeuteropropionic acid, was added at a final concentration of 1 mmol/L, together with 50 μL of ^2^H_2_O, and ^1^H spectra were acquired using a 5-mm ^1^H/broadband inverse detection probe in a 14.1 T NMR spectrometer (Bruker Spectrospin Ltd.) with water pre-saturation and a flip angle of 90° into 16,384 data points, with a spectral width of 7,788 Hz and a repetition time of 8 seconds. Spectra were phased and baseline corrected, and peak integrals calculated using Topspin 4.0.6 (TopSpin, RRID:SCR_014227, Bruker).

### Dynamic contrast-enhanced MRI

A separate cohort of A11, S2, and U87 tumor-bearing animals (3 per group) underwent dynamic contrast-enhanced (DCE)-MRI before and 48 hours after the last radio-chemotherapy treatment. Images were acquired at 9.4 T using a Bruker console, an Agilent magnet and a 40-mm diameter ^1^H volume coil (Agilent). Tumors were localized in axial (FOV, 35×35 mm^2^) and sagittal (FOV, 60×60 mm^2^) ^1^H images acquired with a FSE pulse sequence (TR, 1,000 ms; TE, 25 ms; 256×256 matrix; slice thickness, 1 mm; 10 slices). Baseline and dynamic axial T_1_ images, acquired following tail vein injection of Dotarem, at 200 μmol/kg (Gadoteric acid, Guerbet), were acquired as described previously (20). Signals from the image series were converted, on a pixel-by-pixel basis, to a contrast-agent concentration by assuming an R_1_ relaxivity of the contrast agent of 2.7 s^–1^(mmol/L)^–1^ (29).

### Diffusion-weighted magnetic resonance imaging

Diffusion-weighted ^1^H images were acquired at 9.4 T using a spin echo pulse sequence with echo planar readout (FOV, 40×40 mm^2^; 64×64 matrix; TR, 2 s; TE, 44.86 ms). Tumors were localized in axial and coronal ^1^H images acquired with a FSE pulse sequence (TR, 1,000 ms; TE, 25 ms; FOV, 40×40 mm^2^; 256×256 matrix; slice thickness, 1 mm; 10 slices). Diffusion-sensitizing gradients equivalent to b-values of 16.17, 56.06, 106.06, 206.06, 406.06, and 806.06 s/mm^2^ were applied along the slice axis. The apparent diffusion coefficient (ADC) was measured pretreatment and at 2 and 7 days posttreatment in a 1-mm thick slice covering the tumor.

### Histology and IHC

Brains were immediately transferred to 10% formalin for 24 hours, then 70% ethanol before embedding in paraffin and sectioning (10-μm thick sections). The tissue was cut in the middle of the frontal lobe transversely and stained with hematoxylin and eosin (ST020 Multistainer—Leica Microsystems, RRID:SCR_008960). TUNEL staining was performed using a DeadEnd Colorimetric System Kit (Promega Benelux BV) and cleaved caspase-3 (CC3) staining using Leica's Polymer Refine Kit (Leica Microsystems, catalog no. DS9800, RRID:AB_2891238) on an automated Bond platform (Leica Biosystems Ltd). The antibody was used at a 1:400 dilution (Cell Signaling Technology). Slides were scanned at 20x magnification with a resolution of 0.5 μm per pixel on an Aperio AT2 (Leica Biosystems, RRID:SCR_021256). Images were analyzed using a CytoNuclear v1.6 algorithm on HALO v3.0.311.293 (RRID:SCR_018350, Indica Labs) to quantify percentage of positive cells.

### Statistical analysis

Statistical and graphical analyses were performed using Prism v9 (RRID:SCR_002798, GraphPad). Data are shown as mean ± SD, unless stated otherwise. Analysis of variance was used for multiple comparisons of groups to determine significance. A paired or unpaired Student *t* test was used for single-parameter comparisons. Power calculations were used to define the number of animals required to detect the effect of interest with a probability of 0.9 assuming a familywise error rate of 5%, using the programme G*Power (30). Analysis of recent experiments (31) showed that a group size of 6 to 8 animals would be sufficient to reach this objective, however effect sizes observed here were much larger and we were able to stop at 3 animals per group.

### Data availability

The data generated in this study will be made publicly available in a University of Cambridge Data Repository (https://doi.org/10.17863/CAM.87021).

## Results

### Deuterated fumarate metabolism detects cell death in glioblastoma cells *in vitro*

**Figure 1.**
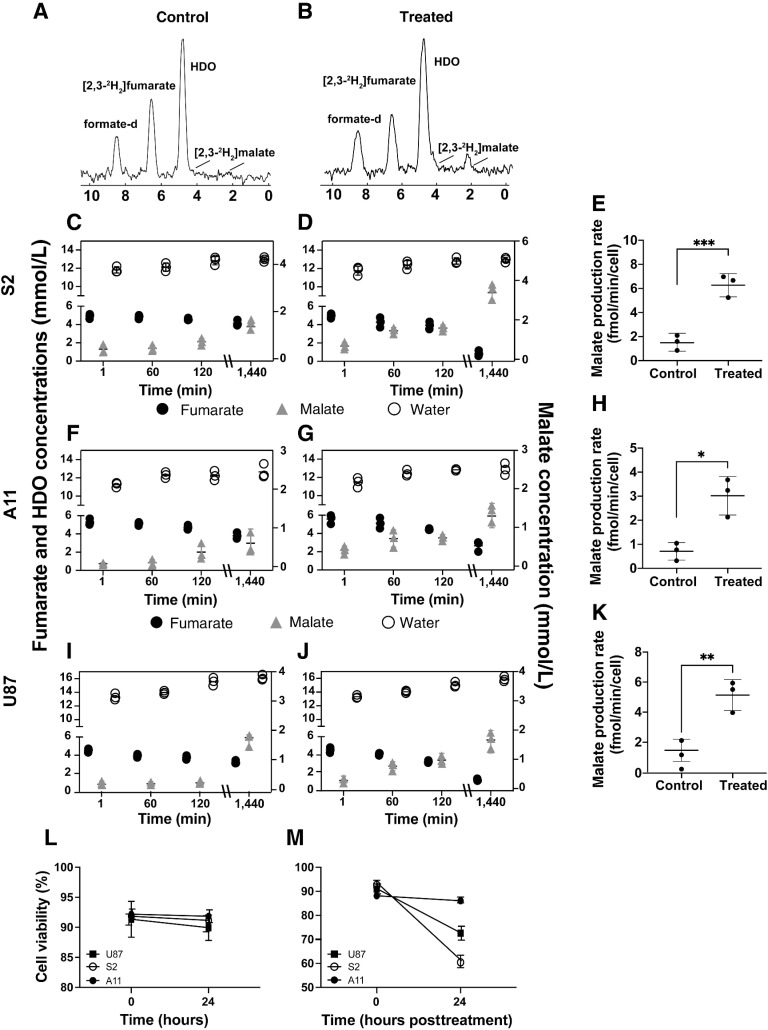
^2^H NMR measurements of labeled fumarate, malate, and water in cell culture medium. **A** and **B,** Representative ^2^H NMR spectra of medium from untreated S2 cells (**A**) and from cells 24 hours after the last chemoradiotherapy treatment (5 Gy per fraction, 15 Gy in total, and 50 μmol/L temozolomide; **B**), 2 hours after the addition of 5 mmol/L [2,3-^2^H_2_]fumarate. Deuterated fumarate, malate, and water concentrations in medium from untreated S2 (**C**), A11 (**F**), and U87 (**I**) cells and treated S2 (**D**), A11 (**G**), and U87 (**J**) cells at the indicated times after addition of 5 mmol/L [2,3–^2^H_2_]fumarate. Rates of labeled malate production in untreated and treated S2 (***, *P* = 0.0028; **E**), A11 (*, *P* = 0.0105; **H**), and U87 (**, *P* = 0.0075; **K**) cell suspensions (mean ± SD; *n* = 3 biological replicates). Cell viability was measured in control (**L**) and chemoradiotherapy (**M**)-treated S2, A11, and U87 cells at the indicated time points following collection of the media samples.

Human glioblastoma (U87) and patient-derived cells (A11, S2) were treated with 50-μmol/L temozolomide and 15 Gy radiation and the concentrations of deuterium labelled water, fumarate and malate were measured following the addition of 5-mmol/L [2,3-^2^H_2_]fumarate using ^2^H NMR measurements on samples of culture medium (Fig. 1A and B). Representative spectra from A11 and U87 cells are shown in Supplementary Fig. S2. There was an increase in the rate of labeled malate production following treatment in all three cell lines. The increase was largest in S2 cells (Fig. 1C–E), consistent with the higher levels of cell death posttreatment, with an increase from 1.84 ± 0.23 fmol/min/cell to 6.76 ± 0.7 fmol/min/cell (*P* = 0.0028; Fig. 1E). The rates were lower in treated A11 (Fig. 1F–H) and U87 cells (Fig. 1I–K), with malate production rates of 3.01 ± 0.79 and 5.13 ± 1.03 fmol/min/cell, respectively compared with rates in untreated controls of 0.71 ± 0.36 and 1.46 ± 0.74 fmol/min/cell (Fig. 1F and I) The rates of water labelling were not significantly affected by the induction of cell death, as observed previously (20). Cell viability was unchanged in the controls (Fig. 1L) but decreased in the treated group (Fig. 1M).

### Deuterated fumarate metabolism detects cell death in glioblastoma tumor models *in vivo*

**Figure 2.**
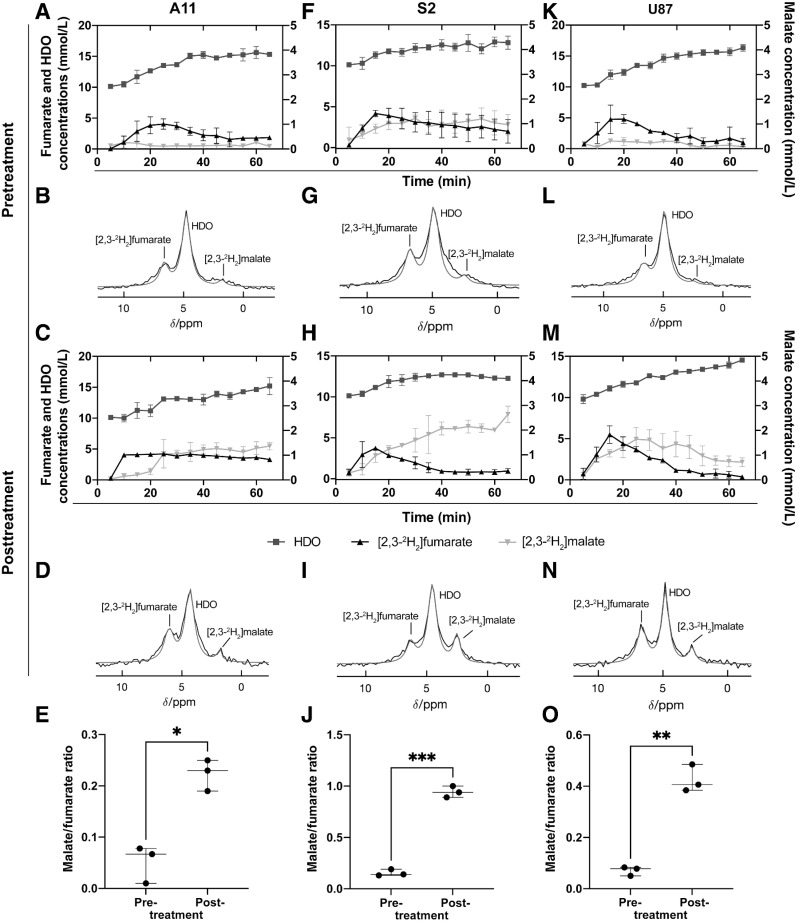
^2^H MR spectroscopy measurements of labeled fumarate, malate, and water concentrations in A11 (**A–E**), S2 (**F–J**), and U87 (**K–O**) tumors. Tumor spectra were acquired before and 48 hours after the last chemoradiotherapy treatment (temozolomide 100 mg/kg, 20 Gy in total, 5 Gy per fraction). Sum of 12 ^2^H spectra recorded over 60 minutes from A11 (**B** and **D**), S2 (**G** and **I**), and U87 (**L** and **N**) tumors. Injection of [2,3-^2^H_2_]fumarate (1 g/kg) started 5 minutes after the start of acquisition of the first spectrum. The peaks were fitted individually using prior knowledge. [2,3-^2^H_2_]malate/[2,3-^2^H_2_]fumarate ratios before and 48 hours after the last chemoradiotherapy treatment of A11 (*n* = 3; *, *P* = 0.0439, paired two-tailed *t* test; **E**), S2 (*n* = 3; ***, *P* = 0.0006, paired two-tailed *t* test; **J**), and U87 (*n* = 3; **, *P* = 0.0061, paired two-tailed *t* test; **O**). Data are presented as mean ± SD.

**Figure 3.**
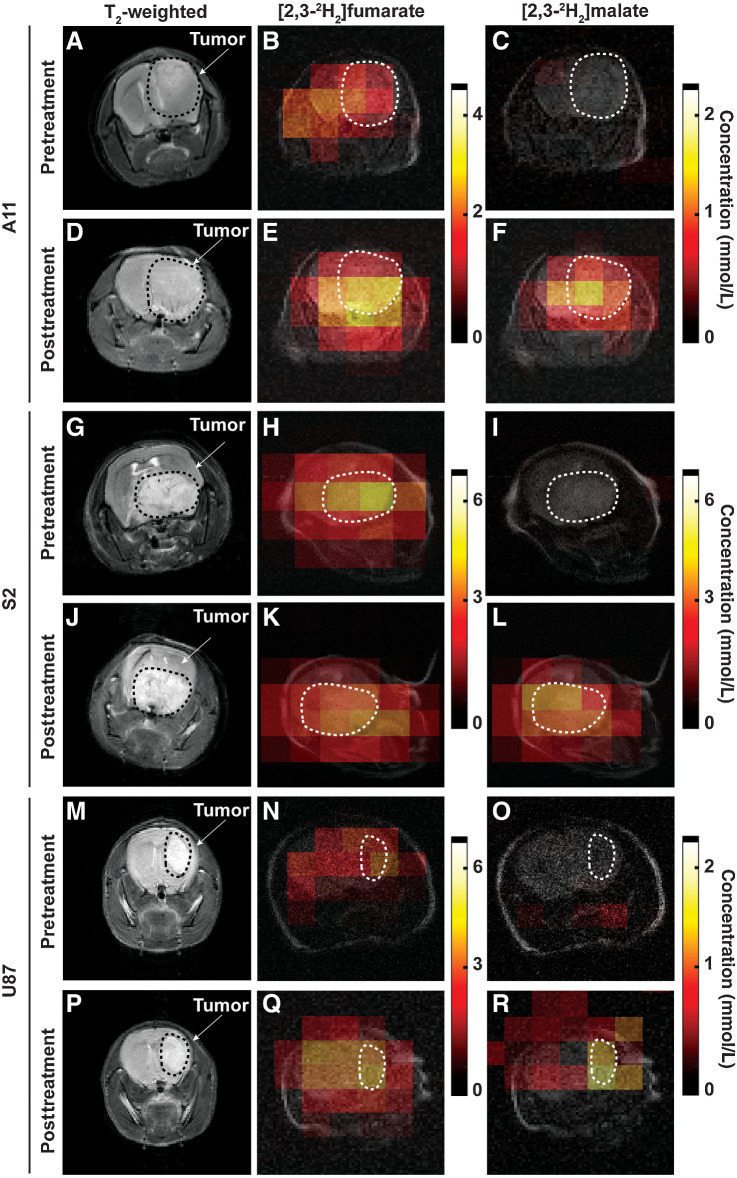
Representative metabolite concentration maps from the central slice derived from a series of dynamic 3D ^2^H CSI images summed over the first 30 minutes of signal acquisition following [2,3-^2^H_2_]fumarate injection into A11 (**B**, **C**, **E**, and **F**), S2 (**H**, **I**, **K**, and **L**), and U87 (**N**, **O**, **Q**, and **P**) tumor-bearing mice. The color code represents concentration (in mmol/L) derived from the ratios of the peak intensities in the malate and fumarate maps to peak intensities in an initial HDO map and corrected for the number of ^2^H labels per molecule and for signal saturation. The locations of the tumors are indicated by dotted white lines determined from an underlying T_2_-weighted ^1^H image acquired concurrently using a ^1^H volume coil. The corresponding T_2_-weighted ^1^H images were acquired separately at 9.4 T. The tumor locations are outlined with dotted black lines. T_2_-weighted ^1^H images (**A**, **G**, and **M**) and the fumarate (**B**, **H**, and **N**) and malate (**C**, **I**, and **O**) concentration maps pretreatment and the corresponding T_2_-weighted ^1^H images (**D**, **J**, and **P**) and the fumarate (**E**, **K**, and **Q**) and malate (**F**, **L**, and **R**) concentration maps 48 hours following the last chemoradiotherapy treatment.

**Figure 4.**
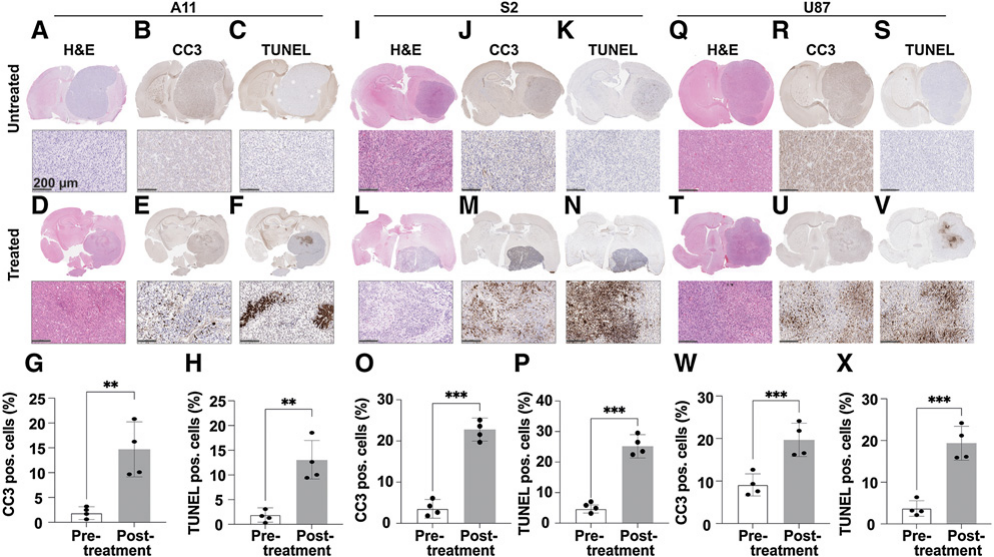
Histologic assessment of tumor cell death 48 hours following chemoradiotherapy treatment. Representative sections of A11 (**A–F**), S2 (**I–N**), and U87 (**Q–V**) tumors stained with hematoxylin and eosin (H&E; **A**, **D**, **I**, **L**, **Q**, and **T**). The top panels show the whole brain section containing the tumor, and the bottom panels show a Multiplication symbol 10 magnification of part of the tumor area. The sections were co-stained for CC3 (**B**, **E**, **J**, **M**, **R**, and **U**) and TUNEL (**C**, **F**, **K**, **N**, **S**, and **V**). The percentage of CC3 (**G**, **O**, and **W**)- and TUNEL (**H**, **P**, and **X**)-positive cells pretreatment and 48 hours after the completion of treatment is shown for A11 (**G** and **H**), S2 (**O** and **P**), and U87 (**W** and **X**) tumors (*n* = 4 per group, drug treated and untreated). Data are shown as mean ± SD. **, *P* < 0.01; ***, *P* < 0.001.

Deuterium-labeled fumarate, malate and water concentrations were monitored following intravenous injection of 1 g/kg [2,3-^2^H_2_]-disodium fumarate, using localized ^2^H spectroscopy (Fig. 2) and imaging (Fig. 3). The tumor fumarate concentrations were similar in all three tumor models before (Fig. 2A, B, F, G, K, and L) and at 48 hours after completion of chemoradiotherapy (Table 1; Fig. 2C, D, H, I, M, and N). However, all showed a significant increase in labeled malate concentration following treatment, which in the A11 and S2 tumors was confirmed by measurements in tumor extracts (Table 1; Supplementary Fig. S3). In extracts of untreated tumors the concentration of [2,3-^2^H_2_]malate, determined by ^2^H NMR, was only 29% (A11) and 30% (S2) of the unlabeled concentration, determined by ^1^H NMR, whereas in treated tumors the total malate concentration increased by ∼3.2 and ∼4.4x, of which, ∼36% (A11) and 44% (S2) were deuterium labeled (Table 1). The malate/fumarate signal ratios measured 48 hours after the last treatment increased significantly in all tumor models, with S2 showing the largest increase (Fig. 2E, J, and O). A fast, dynamic 3D CSI sequence showed no detectable malate signal pretreatment (Fig. 3C, I, and O) whereas increased malate concentrations were observed 48 hours after the last treatment in all tumor models (Fig. 3F, L, and R), with the highest concentration in S2 tumors (Fig. 3L). The tumor malate signal-to-noise ratios posttreatment were 2.05 ± 0.27, 5.64 ± 2.54, and 5.38 ± 2.62 in A11, S2, and U87, respectively and 1.29 ± 0.11, 4.01 ± 2.98, and 3.67 ± 1.12 in the corresponding CSI (data are expressed as mean ± SD, *n* = 3 for the spectroscopy data and *n* = 2 for the images). Representative spectra from the 3D CSI dataset are shown in Supplementary Fig. S4. The higher rates of malate production in S2 cells *in vitro* and S2 tumors *in vivo* when compared with the other cell lines cannot be explained by higher levels of fumarase activity in these cells, which was similar in all three lines, both in cells in culture and tumors *in vivo* (Supplementary Fig. S5). Treatment slowed the growth of all three tumors, which was significant for S2 and U87 tumors (Supplementary Fig. S6; Supplementary Table S1). The greater inhibition of S2 tumor growth was correlated with increased survival (Supplementary Fig. S7), although animals with the other tumors also showed evidence of increased survival. Increases in the [2,3-^2^H_2_]malate/[2,3-^2^H_2_]fumarate signal ratios were correlated with the levels of tumor cell death, as determined from histologic measurements of CC3 and DNA damage (TUNEL) in tumor sections (Fig. 4). Although, there were no decreases in tumor size following treatment there were significant increases in CC3 and TUNEL staining. CC3 staining increased from 2.84 ± 1.29 to 11.66 ± 3.71 in A11 tumors (*P* = 0.002, *n* = 4; Fig. 4G), from 9.10 ± 2.60 to 19.67 ± 3.91 in U87 tumors (*P* = 0.004, *n* = 4; Fig. 4W) and showed the greatest increase in S2 tumors, from 3.52 ± 2.27 to 22.78 ± 2.78 (*P* = 0.0008, *n* = 4; Fig. 4N). There were parallel increases in TUNEL staining, which increased from 2.38 ± 1.44 to 10.91 ± 3.85, from 4.15 ± 1.33 to 25.17 ± 3.82 and from 3.70 ± 1.86 to 19.35 ± 4.05 in A11 (*P* = 0.0035, *n* = 4; Fig. 4H), S2 (*P* = 0.0009, *n* = 4; Fig. 4O), and U87 (*P* = 0.0004, *n* = 4; Fig. 4X) tumors, respectively.

**Table 1.** Deuterium-labelled fumarate, malate, and water concentrations measured in the indicated tumor extracts and in extracts of the corresponding contralateral hemisphere using ^2^H NMR.

	Untreated		Treated	
	Concentrations of deuterated species (μmol/g)			
	[2,3-^2^H_2_]fumarate	[2,3-^2^H_2_]malate	[2,3-^2^H_2_]fumarate	[2,3-^2^H_2_]malate
A11 Tumor	5.06 ± 1.12	0.09 ± 0.23	4.79 ± 1.75	0.45 ± 0.41
CTL	0.83 ± 0.35	0.11 ± 0.07	1.04 ± 0.53	0.19 ± 0.12
S2 Tumor	5.16 ± 1.56	0.12 ± 0.09	5.47 ± 2.02	1.02 ± 0.33
CTL	0.62 ± 0.23	n.d.	1.41 ± 0.31	0.27 ± 0.11

	Concentrations of protonated species (μmol/g)			
	Fumarate	Malate	Fumarate	Malate
A11 Tumor	1.81 ± 0.82	0.31 ± 0.16	1.76 ± 1.72	0.81 ± 0.17
CTL	0.52 ± 0.31	0.16 ± 0.06	0.62 ± 0.14	0.19 ± 0.06
S2 Tumor	1.63 ± 0.25	0.40 ± 0.17	1.87 ± 1.13	1.29 ± 0.24
CTL	0.45 ± 11	0.36 ± 0.09	0.77 ± 0.26	0.25 ± 0.21

The concentrations of the protonated species were measured using ^1^H NMR. The protonated malate concentration was corrected for background signal at ∼2.4 ppm, which was determined by acquiring spectra from mice that had not been injected with [2,3-^2^H_2_]fumarate prior to tissue collection. For [2,3-^2^H_2_]malate this was based on the upfield ^2^H resonance at 2.4 ppm, the downfield resonance was not resolved from the water resonance. The tissues were extracted at 20 min after intravenous injection of [2,3-^2^H_2_]fumarate. Data are expressed as mean ± SD (*n* = 3).

Abbreviation: CTL, contralateral hemisphere; n.d., not detected.

### Detection of treatment response using hyperpolarized [1,4-^13^C_2_, 2,3-^2^H_2_]fumarate

**Figure 5.**
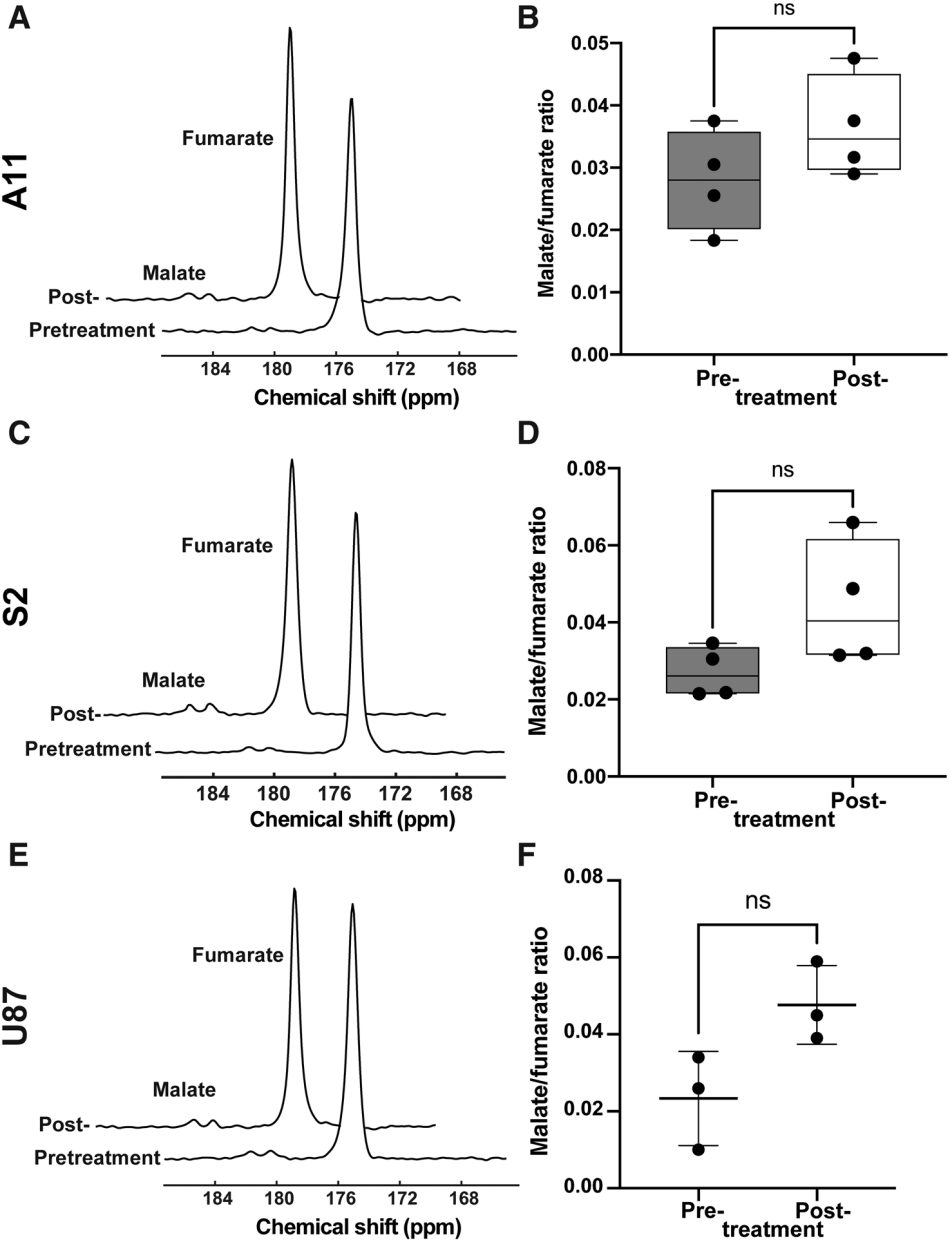
^13^C MR spectra acquired following intravenous injection of hyperpolarized [1,4-^13^C_2_,2,3-^2^H_2_]fumarate. Summed spectra showing flux of hyperpolarized ^13^C label between [1,4-^13^C_2_,2,3-^2^H_2_]fumarate (177.2 ppm) and [1,4-^13^C_2_,2,3-^2^H_2_]malate (182.2, 183.6 ppm) were acquired from an 8-mm tumor slice over a period of 180 seconds starting from the time of injection. Spectra were acquired from A11 (**A**), S2 (**C**), and U87 (**E**) tumors before and at 48 hours after the last chemo-radiotherapy session. Malate/fumarate signal ratios in A11 (*n* = 4, paired two-tailed *t* test; **B**), S2 (*n* = 4, paired two-tailed *t* test; **D**), and U87 (*n* = 3, paired two-tailed *t* test; **F**) tumors pre- and posttreatment. The ratios were obtained from the summed spectra. ns, nonsignificant.

The hyperpolarized [1,4-^13^C_2_, 2,3-^2^H_2_]malate/[1,4-^13^C_2_, 2,3-^2^H_2_]fumarate signal ratio (the ratio of the areas under the malate and fumarate labelling curves) increased in all three tumor models following treatment (Fig. 5). However, none of these increases reached statistical significance; with increases of 33.3 ± 1.8% (*P* = 0.125, *n* = 4) in A11 (Fig. 5B), 62.9 ± 14.6% in S2 (*P* = 0.133, *n* = 4; Fig. 5D) and 51.1 ± 16.2% in U87 (*P* = 0.08, *n* = 3) tumors (Fig. 5F). The malate signal-to-noise ratio (SNR) in the posttreatment spectra was 3.26 ± 2.54, 4.12 ± 2.192, and 4.44 ± 2.07 in A11, S2, and U87 tumors, respectively. These experiments were performed following the ^2^H measurements, when at least in U87 tumors the [1,4-^13^C_2_, 2,3-^2^H_2_]fumarate had cleared by 65 minutes (Fig. 2). The animals used in the ^2^H, ^13^C, dynamic contrast agent-enhanced and diffusion-weighted imaging experiments are summarized in Supplementary Table S2. ^13^C CSI are shown in Supplementary Fig. S8.

### Dynamic contrast agent–enhanced MRI measurements

**Figure 6.**
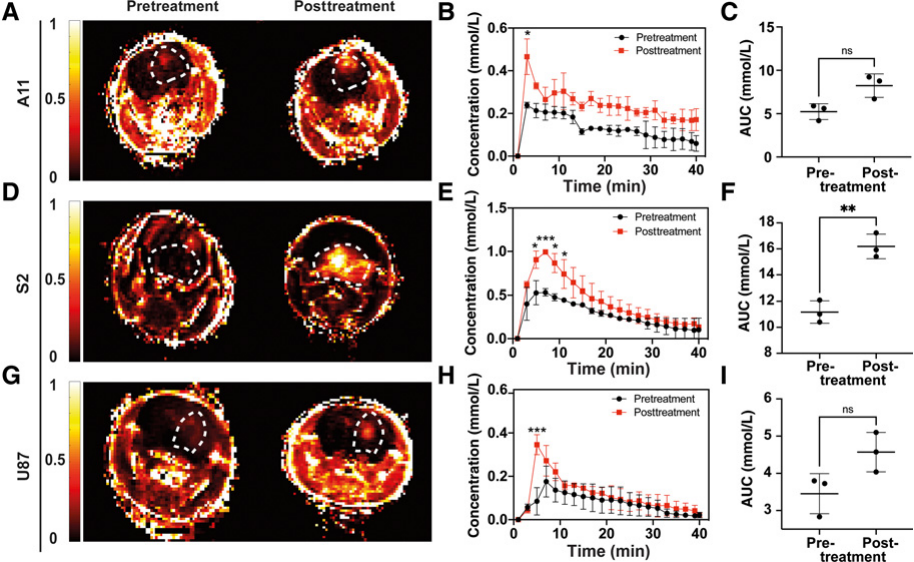
DCE MRI measurements of perfusion in A11 (*n* = 3; **A–C**), S2 (*n* = 3; **D–F**), and U87 (*n* = 3; **G–I**) tumors before and after treatment. Representative color intensity maps of mean contrast agent concentration over the first three minutes following injection in A11 (**A**), S2 (**D**), and U87 (**G**) tumors. The locations of the tumors are outlined by dotted white lines. Estimated contrast agent concentration in tumor tissue at the indicated times after intravenous injection before and 48 hours following the last chemoradiotherapy treatment of A11 (**B**), S2 (**E**), and U87 (**H**) tumors. Data are shown as mean ± SD; *, *P* < 0.05; ***, *P* < 0.001. The area under the uptake curve (AUC) showed an increase in contrast agent uptake in S2 tumors (**, *P* = 0.0024, paired two-tailed *t* test) following treatment (**F**), while there were no significant differences in A11 (**C**) and U87 (**I**) tumor-bearing animals. Data are presented as mean ± SD. ns, nonsignificant.

Changes in tumor contrast agent uptake were assessed in separate cohorts of A11 (*n* = 3), S2 (*n* = 3), and U87 (*n* = 3) tumor-bearing mice (Fig. 6). The area under the tumor contrast agent uptake curve increased significantly in S2 tumors at 48 hours posttreatment (Fig. 6D–F) but not in A11 (Fig. 6A–C) or U87 tumors (Fig. 6G–I), although all three tumors showed higher tumor contrast agent concentrations posttreatment with significantly higher concentrations in the first 10 minutes post contrast agent injection (Fig. 6B, E, and H).

### Diffusion-weighted MRI measurements

**Figure 7.**
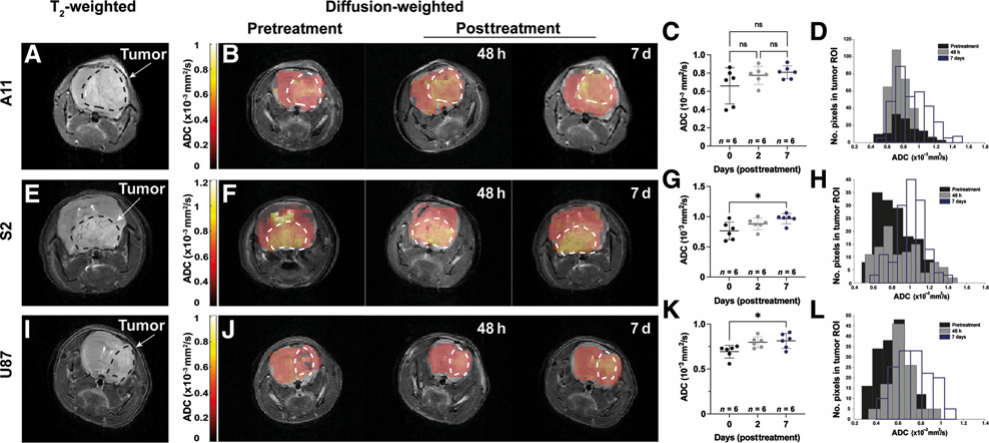
Diffusion-weighted imaging of A11, S2, and U87 tumors before and after treatment. Representative T_2_-weighted axial slices from reference ^1^H images acquired at 9.4 T from an A11 tumor (**A**), S2 tumor (**E**), and U87 tumor posttreatment (**I**) at 7 days after treatment. The locations of the tumors are outlined by dotted black lines. Representative diffusion images, in false color, overlaid on the corresponding T_2_-weighted images acquired from A11 (**B**), S2 (**F**), and U87 (**J**) tumors before and at 48 hours and 7 days after the last chemoradiotherapy treatment. The locations of the tumors are outlined by dotted white lines. The underlying T_2_-weighted images acquired at 7 days after treatment are the same as those shown in **A**, **E**, and **I** and the underlying image shown in **F** at 48 hours posttreatment is the same as that shown in Fig. 3J. The ^2^H and diffusion-weighted images were acquired in the same imaging session as the T_2_-weighted images. Mean ADC measured along the slice-selective direction before and at 2 and 7 days after the last chemoradiotherapy treatment in A11 (*n* = 6; **C**), S2 (*n* = 6; **G**), and U87 (*n* = 6; **K**) tumors. Histograms of ADC values over all the animals imaged in A11 (**D**), S2 (**H**), and U87 (**L**) tumors. *, *P* < 0.05; ns, nonsignificant.

The mean ADC of tumor water, measured using diffusion-weighted MRI (DW-MRI; Fig. 7), was unchanged at 48 hours after the last chemoradiotherapy treatment in all tumor models (Fig. 7C, G, and K) and the ADC maps remained largely homogeneous (Fig. 7D, H, and L). At 7 days after treatment, however, the S2 and U87 tumors showed a significant increase in the mean ADC (Fig. 7G and K).

## Discussion

Routine MRI measurements of glioblastoma morphology are unable to distinguish reliably between true disease progression and pseudoprogression, which has prompted a search for MRI methods that are better able to discriminate between the two (32).

Tumor water ADC has been reported to be lower in true disease progression as compared with pseudoprogression, however meta-analyses have shown that while diagnostic performance was superior to that of conventional anatomic imaging it was poorer when compared with MRI measurements of perfusion (13). Dynamic susceptibility contrast (DSC) MRI measurement of relative cerebral blood volume (rCBV) is the most widely used perfusion technique, with numerous studies showing that rCBV is lower in areas of radionecrosis and pseudoprogression when compared with true disease progression. DCE MRI measurements of contrast agent leakage from the tumor vasculature, characterized by a transfer constant (Ktrans), which is a function of vessel permeability, vessel surface area and blood flow, can also distinguish true progression from pseudoprogression, where Ktrans is lower in regions of radionecrosis and pseudoprogression. However, with no widely established Ktrans thresholds and a dependence of the fitted parameter on the model used to analyze the data this is a less widely used technique than DSC MRI (33). Imaging of proton transfer between protein amide groups and water has shown promise in distinguishing true progression from pseudoprogression, with an almost two-fold higher rate in the case of true progression (34), although currently the technique has not been as widely evaluated as the other MRI methods. ^1^H MRS measurements of tumor metabolism can distinguish between true progression and pseudoprogression, with elevated choline concentrations indicating increased tumor cell density and progression and posttreatment necrotic tumors showing elevated lipid and lactate peaks and lower choline concentrations (32, 35–38). However, while meta-analysis has shown that ^1^H MRS is superior to anatomic MRI, DWI-MRI, and MRI-based perfusion measurements in distinguishing true progression from pseudoprogression (13) the low signal-to-noise ratio in spectroscopy means that scan times are long.

The problem of low sensitivity of MRS measurements of tumor metabolism has been addressed using nuclear spin hyperpolarization, which can result in a massive increase in sensitivity (39). We have shown previously that imaging the metabolism of hyperpolarized [1-^13^C]pyruvate can detect the response of the A11 PDX used here to chemoradiation by 72 hours after treatment, in an image that took less than 30 seconds to acquire (identified as GB4 in this previous paper; ref. 40). However, the decrease in lactate labeling from the injected pyruvate was relatively small (∼29%) and much less than the increase in the [2,3-^2^H_2_]malate/[2,3-^2^H_2_]fumarate signal ratio observed here at 48 hours after treatment despite a similar increase in cell death. The [2,3-^2^H_2_]malate/[2,3-^2^H_2_]fumarate ratio increased in A11 tumors by 340%, in the more radiosensitive S2 tumors by 755%, and in the U87 cell line model by 425%. The increased rate of malate production in S2 tumors cannot be explained by intrinsically higher levels of fumarase activity, which was similar in all three tumor models. The increased rates in A11 and S2 tumors also cannot be explained by increased fumarate delivery because the concentrations of fumarate in the tumors pre- and posttreatment were similar. Moreover, by using the [2,3-^2^H_2_]malate/[2,3-^2^H_2_]fumarate ratio as an indicator of cell death the effects of any possible changes in fumarate delivery are corrected for. We have shown previously, in three drug-treated tumor models, that the accumulation of malate in a tumor posttreatment cannot be explained by metabolism of fumarate elsewhere in the body and wash in of labeled malate (20).

The increases in the hyperpolarized [1,4-^13^C_2_, 2,3-^2^H_2_]malate/[1,4-^13^C_2_, 2,3-^2^H_2_]fumarate signal ratio following treatment, following injection of hyperpolarized [1,4-^13^C_2_, 2,3-^2^H_2_]fumarate, were much smaller and did not reach statistical significance. This is a reflection of the short half-life of the hyperpolarized ^13^C label, which means that labeled malate accumulation can only be measured over a few minutes whereas in the ^2^H experiments labeled malate accumulation was measured over a much longer period of up to 65 minutes. This is demonstrated by the cell experiments, where the 2,3-^2^H_2_]malate/[2,3-^2^H_2_]fumarate ratio was 0.03 to 0.04 pretreatment and posttreatment ranged from 0.07 to 0.14 at 3 minutes and from 0.3 to 0.5 at 60 minutes. The relative increases in these ratios are similar to those observed in the hyperpolarized ^13^C experiments *in vivo* at 3 minutes and in the ^2^H experiments *in vivo* at 60 minutes.

Detection of treatment response using [2,3-^2^H_2_]fumarate was also more sensitive than either of the more advanced imaging techniques used currently to distinguish between true disease progression and pseudoprogression. DW-MRI detected treatment response only in S2 and U87 tumors, which showed higher levels of cell death posttreatment than A11 tumors, and then only at 7 days posttreatment. We have shown previously that both ^2^H- (20) and ^13^C-labeled (17) fumarate are more sensitive than DW-MRI at detecting low levels of diffuse necrosis. Dynamic contrast agent enhanced images showed an increase in the area under the contrast agent uptake curves in all three tumor models at 48 hours posttreatment but this was only significant in the S2 tumors, which showed the highest levels of cell death posttreatment.

Early detection of glioblastoma cell death following chemo-radiation has the potential to distinguish true disease progression from pseudoprogression in the clinic. We have shown here that ^2^H MRI of labeled malate production from injected [2,3-^2^H_2_]fumarate can detect cell death in two orthotopically implanted PDX models and a cell line model of glioblastoma within 48 hours of treatment and that it is a more sensitive method of detecting cell death than two MRI methods that have been used in the clinic to distinguish between true progression and pseudoprogression. The clinical feasibility of the technique can be assessed from the measured fumarate and malate concentrations in the tumor, which is more appropriate than comparing SNR because the latter depends not only on magnetic field strength, but also on other factors, such as image resolution and coil performance. De Feyter and colleagues (41) detected 1.5 to 2 mmol/L ^2^H-labelled glucose and glutamate/glutamine in the human brain at 4 T. Here we measured deuterated fumarate and malate concentrations of up to 5 mmol/L. Therefore, even with a ∼40% decrease in SNR at 3T (42), these concentrations coupled with the chemical shift separation of the resonances (20), suggests that this method could be used clinically.

## Supplementary Material

Supplementary Data
