# Supplementary material for: Imaging Glioblastoma Response to Radiotherapy Using 2H Magnetic Resonance Spectroscopy Measurements of Fumarate Metabolism
Source: Cancer Res. 2022 Aug 16;82(19):3622–33. doi: 10.1158/0008-5472.CAN-22-0101 (PMC9530651; doi:10.1158/0008-5472.CAN-22-0101)
Supplement: Supplementary Data [file can-22-0101_supplementary_data_suppsmst1-st2sf1-sf8.docx]

**SUPPLEMENTARY INFORMATION**

**­Imaging response of glioblastoma patient-derived xenografts to radiotherapy using ^2^H MRI measurements of [2,3-^2^H_2_]fumarate metabolism**

Friederike Hesse^1^, Alan J. Wright^1^, Vencel Somai^1,2^, Flaviu Bulat^1, 3^, Felix Kreis^1^, Kevin M. Brindle^1,4^

^1^Cancer Research UK Cambridge Institute, University of Cambridge, Li Ka Shing Centre, Robinson Way, Cambridge, United Kingdom.

^2^Department of Radiology, University of Cambridge, School of Clinical Medicine Box 218, Cambridge Biomedical Campus

^3^Department of Chemistry, University of Cambridge,  Lensfield Road, Cambridge, United Kingdom

^4^Department of Biochemistry, University of Cambridge, Tennis Court Road, Cambridge, United Kingdom.

**Supplementary Methods**

**Measurements of fumarase activity in tissue extracts**

Fumarase activity was determined using a colorimetric kit (ab196992, Abcam).  Untreated tumors were freeze-clamped and homogenized in assay buffer using a Precellys 24 homogenizer (Stretton Scientific). The enzyme activity was assayed spectrophotometrically by measuring the conversion of malate to fumarate from the increase in absorbance at 450 nm, according to the manufacturer’s instructions. The measurements were made using a PHERAstar FS microplate reader (BMG Labtech). Protein concentrations were measured using a Direct Detect spectrometer (Merck).

**^13^C spectroscopic imaging**

A FID-CSI sequence was used with a 6 mm slice thickness and  2 spatial dimensions acquired as a centre out spiral of phase encodes, covering a 16 x 16 matrix. A 6010Hz spectral window was acquired encoded by 256 points and a repetition time of 47 ms to give a total scan time of 12.5 s. The pulse sequence was initiated 20 s after an intravenous bolus of [1,4-^13^C_2_,2,3-^2^H_2_] fumarate was administered.

**Supplementary Table 1**

Tumor volumes at baseline (0) and at 2 and 7 days post-treatment were estimated from multi-slice T_2_-weighted images as described in the legend to Supplementary Figure 6. Data were acquired from the cohorts used to study survival (see Supplementary Figure 7). Tumor volumes are shown in mm^3^ (mean ± SD n=8 (A11, S2) and n=6 (U87) biological replicates).

| **Days post-treatment** | **Untreated** | | | **Treated** | | |
| --- | --- | --- | --- | --- | --- | --- |
|  | A11 | S2 | U87 | A11 | S2 | U87 |
| 0 | 68.8 ± 3.9 | 66.8 ± 4.5 | 72.3 ± 3.6 | 69.8 ± 2.2 | 68.7 ± 4.7 | 78.8 ± 3.8 |
| 2 | 83.0 ± 4.5 | 76.9 ± 3.5 | 107.7 ± 3.6 | 79.5 ± 1.8 | 72.5 ± 3.4 | 95.1 ± 7.0 |
| 7 | 92.8 ± 3.3 | 85.6 ± 4.5 | 210.9 ± 5.1 | 86.3 ± 4.4 | 75.9 ± 4.4 | 158.9 ± 9.6 |

**Supplementary Table 2**. Assignment of animals to imaging experiments.

| Animal | DWI | DCE | ^13^C spectroscopy | ^13^C spectroscopic imaging | ^2^H spectroscopy | ^2^H spectroscopic imaging |
| --- | --- | --- | --- | --- | --- | --- |
| A11 | | | | | | |
| 21259 |  |  |  | Yes |  | Yes |
| 27984 | Yes |  |  |  |  |  |
| 27986 |  |  | Yes |  | Yes |  |
| 27987 |  |  | Yes |  | Yes |  |
| 27988 |  |  | Yes |  |  | Yes |
| 21249 |  |  | Yes |  | Yes |  |
| 21251 | Yes |  |  |  |  |  |
| 21253 | Yes | Yes |  |  |  |  |
| 21260 | Yes | Yes |  |  |  |  |
| 21259 | Yes |  |  |  |  |  |
| 21263 | Yes | Yes |  |  |  |  |
| S2 | | | | | | |
| 27979 |  |  | Yes |  | Yes |  |
| 27980 |  |  |  | Yes |  | Yes |
| 27981 |  |  | Yes |  | Yes |  |
| 27983 | Yes |  |  |  |  |  |
| 21254 |  |  | Yes |  | Yes |  |
| 21255 | Yes |  |  |  |  |  |
| 21256 | Yes | Yes |  |  |  |  |
| 21257 |  |  | Yes |  |  | Yes |
| 21258 | Yes |  |  |  |  |  |
| 18982 | Yes | Yes |  |  |  |  |
| 18983 | Yes | Yes |  |  |  |  |
| 18984 | Yes |  |  |  |  |  |
| U87 | | | | | | |
| 21263 |  |  |  | Yes |  | Yes |
| 21264 |  |  |  |  |  | Yes |
| 21265 |  |  | Yes |  | Yes |  |
| 21266 |  |  | Yes |  | Yes |  |
| 21267 |  |  | Yes |  | Yes |  |
| 18979 | Yes |  |  |  |  |  |
| 17850 | Yes | Yes |  |  |  |  |
| 17851 | Yes |  |  |  |  |  |
| 17874 | Yes | Yes |  |  |  |  |
| 17848 | Yes | Yes |  |  |  |  |
| 03033 | Yes |  |  |  |  |  |

**Supplementary Figures**


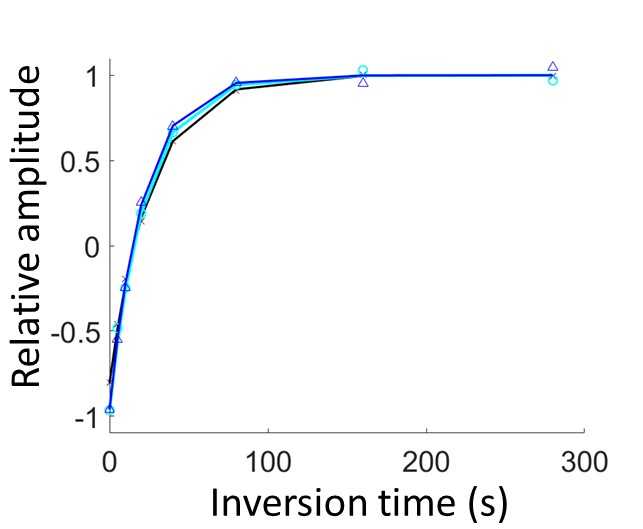


**Supplementary Figure 1**. An inversion recovery sequence was used to measure the T_1_ of 33 mM [1,4-^13^C,2,3-^2^H_2_]fumarate in PBS at pH 7. The measurement was made at 7 T with a 3 cm diameter transmit/receive surface coil tuned to a ^13^C frequency of 75.4 MHz. The relative peak amplitudes were plotted at various inversion times and the line shows a fit to the function S=1-A*exp(-TI/T_1_) where S is signal amplitude, A is a variable to account for imperfect inversion and TI is the inversion time. The T_1_ from 3 separate measurements was found to be 23.2 ± 2.5 s S.D. (n=3).

.

**
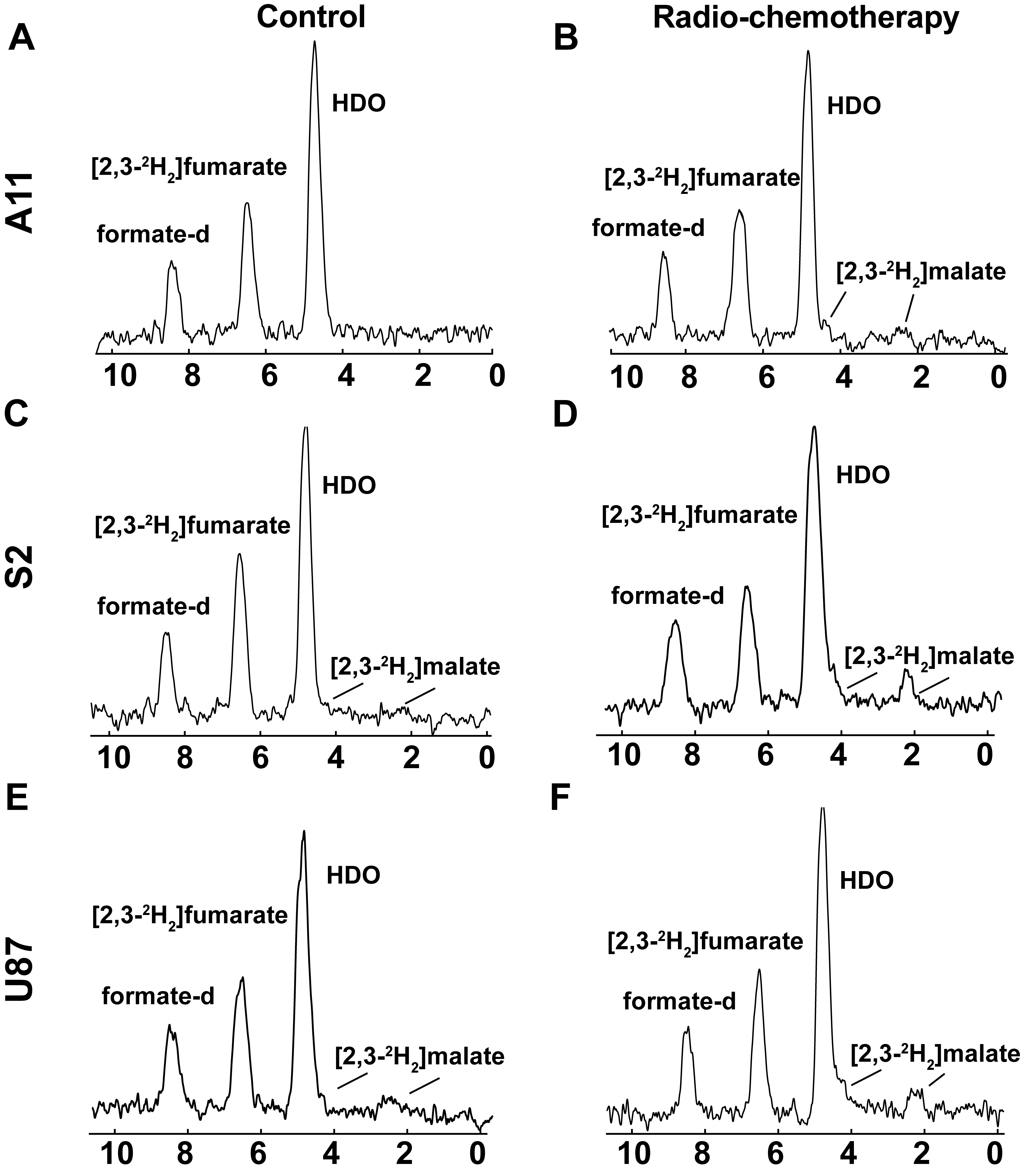
**

**Supplementary Figure 2.** ^2^H NMR measurements of labeled fumarate, malate and water in cell culture medium. ^2^H NMR spectra of medium from untreated A11 (**A**), S2 (**C**) and U87 (**E**) cells and the same cells (**B**, **D**, **F**) following treatment for three consecutive days with a total of 15 Gy of radiation and 50 µM temozolomide. The spectra were acquired 24 h after the last treatment and 2 h after the addition of 5 mM [2,3-^2^H_2_]fumarate.

**­­­
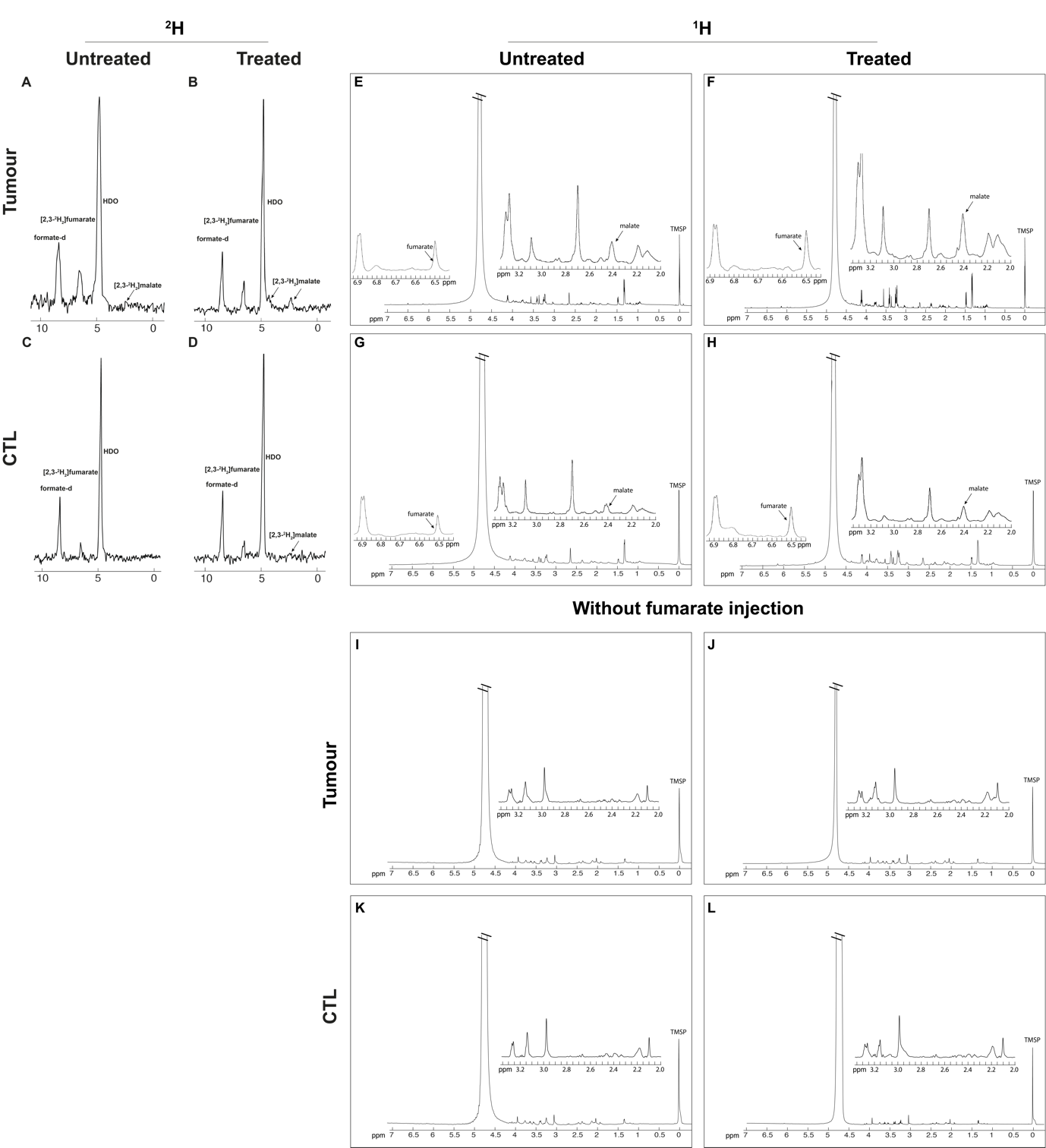
**

**Supplementary Figure 3.** Representative ^2^H (**A-D**) and ^1^H (**E-H**) spectra of fumarate, malate and water (HDO) in S2 tumor extracts and in extracts of the corresponding contralateral hemisphere (CTL). The tissues were extracted at 20 min after i.v. injection of [2,3-^2^H_2_]fumarate. Representative ^1^H spectra (**I-L**) of S2 tumor extracts and extracts of the corresponding contralateral hemisphere (CTL) obtained from animals that had not been injected with fumarate. There was little signal in the region of the malate resonance at 2.4 ppm in these extracts. Subtraction of the average signal intensity in this region of the spectrum from the spectra of extracts from animals injected with fumarate resulted in a minimal correction of the estimated malate concentration. TMSP, 3-(trimethylsilyl)-2,2,3,3-tetradeuteropropionic acid.

**
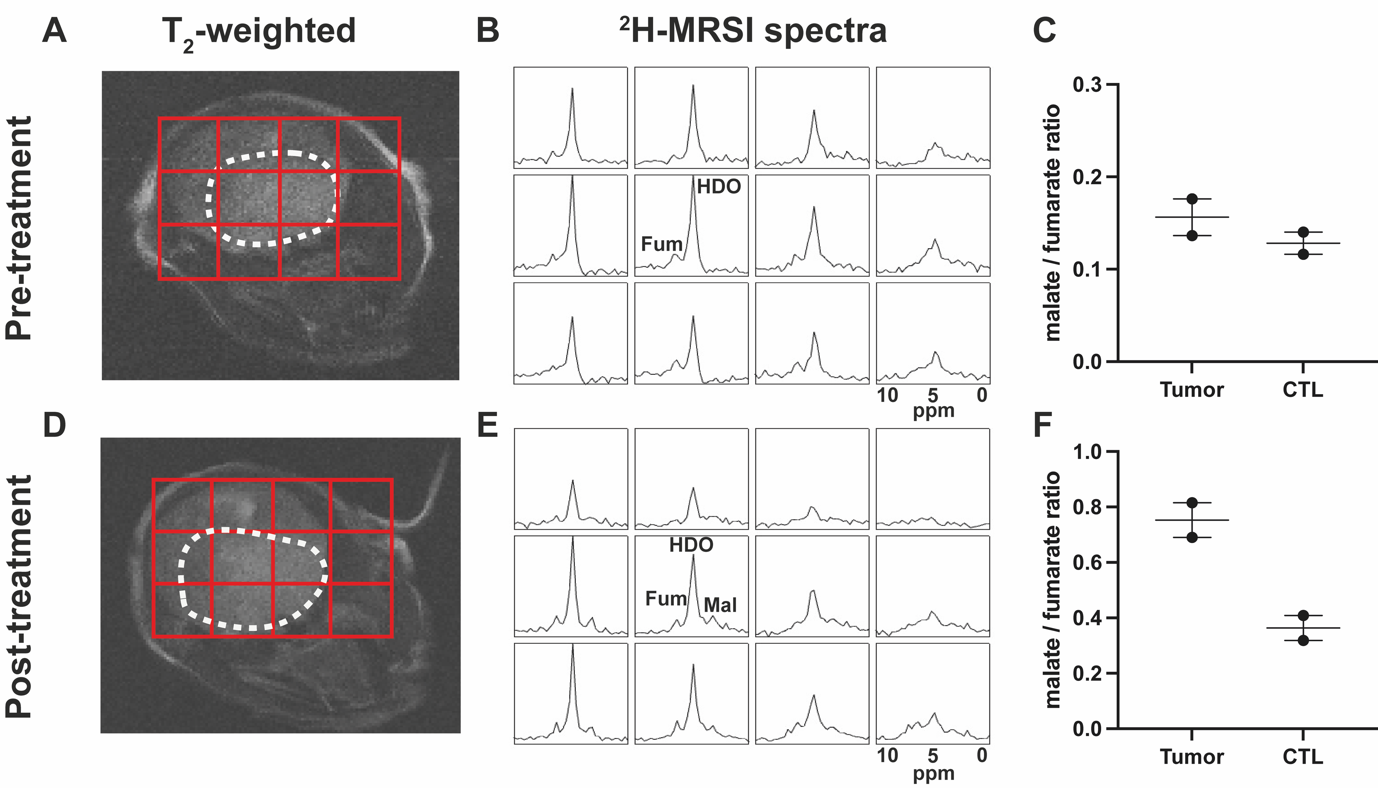
**

**Supplementary Figure 4.** (**A**) T_2_-weighted ^1^H image of a mouse brain with an S2 tumor indicating the positions of the ^2^H MRSI voxels shown in (**B**). The tumor is outlined with a white dotted line and the voxel locations are indicated by the red boxes. **B**) ^2^H spectra from a 3D data set. The spectra correspond to the false color images shown in Figure 3. The [2,3-^2^H_2_]fumarate (Fum), [2,3-^2^H_2_]malate (Mal) and deuterated water (HDO) resonances are indicated. (**D**) T_2_-weighted ^1^H image of the mouse brain with an S2 tumor shown in (**A**) following treatment. The positions of the ^2^H MRSI voxels shown in (**E**) are indicated. The malate/fumarate ratio was higher in the treated tumor (**F**) when compared to the untreated tumor (**C**) and higher than in the contralateral hemisphere (**F**).

**
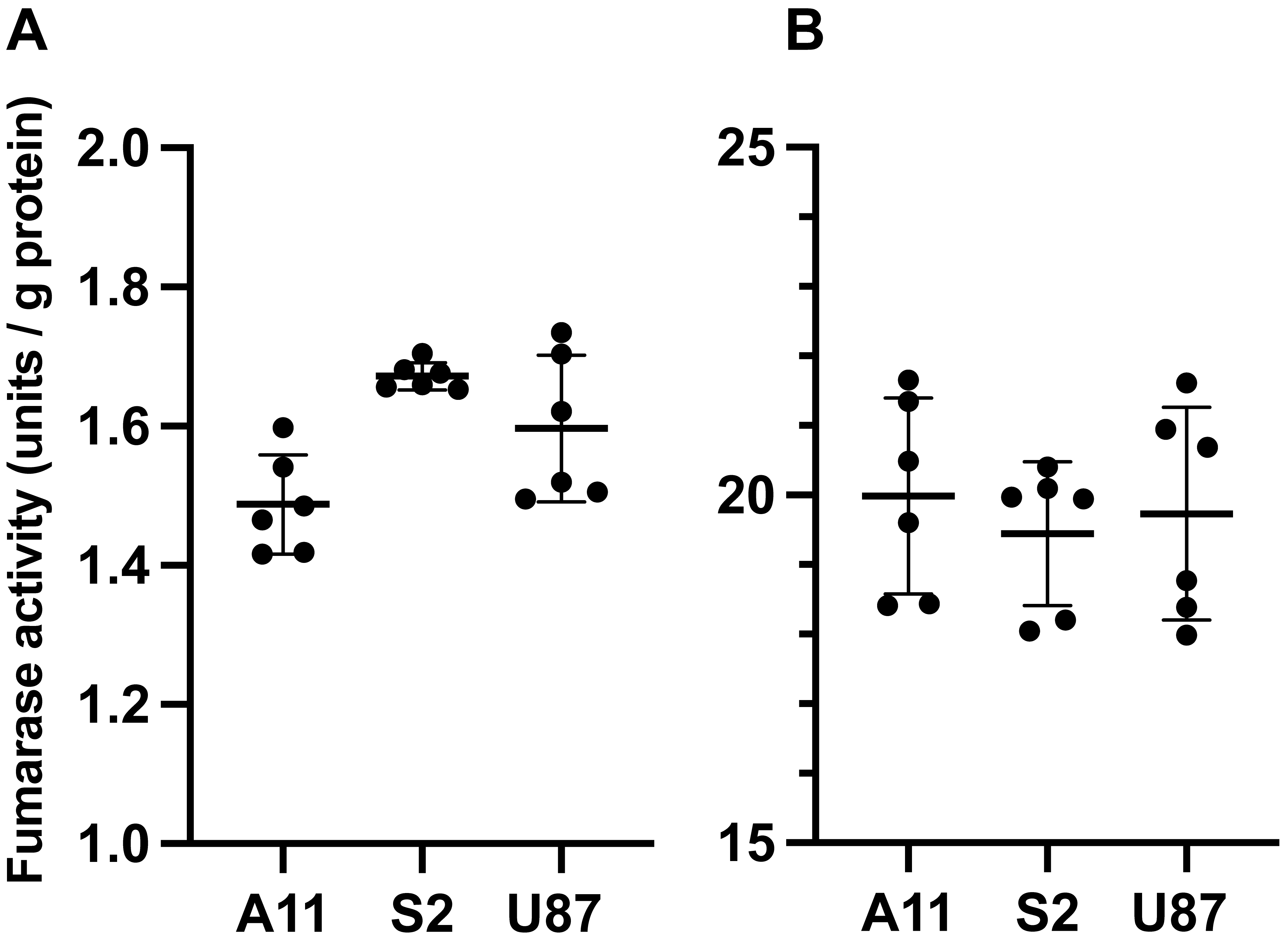
**

**Supplementary Figure 5.** Fumarase activity in A11, S2 and U87 cells (**A**) and tumor extracts (**B**). Data are presented as mean ± SD.


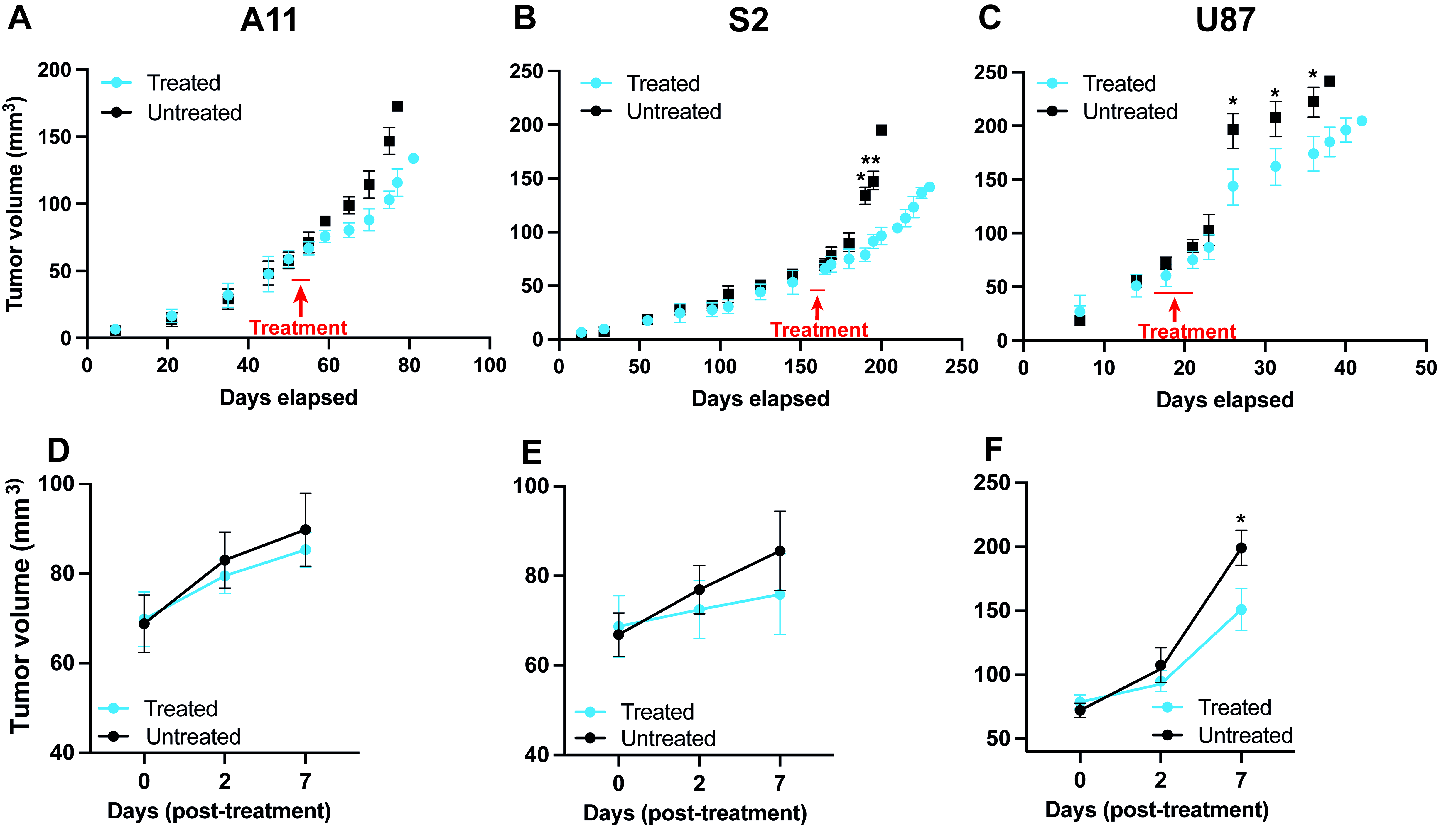


**Supplementary Figure 6.** Tumor volumes calculated from T_2_-weighted ^1^H images. This study used a separate cohort of animals to those used for the imaging studies summarized in Supplementary Table 2. Images were acquired with a 40 x 40 mm field of view into 256 x 256 data points and a slice thickness of 1 mm. The tumor area was circled on successive axial slices and the total volume of the tumor in the slice calculated by multiplying the sum of the voxels by 1 x (40/256)^2^ = mm^3^. The total volume of the tumor was calculated by adding the tumor volumes from all those slices that contained tumor (mean ± SD n=8 (A11, S2) and n=6 (U87) biological replicates). Volumes of A11 (**A**), S2 (**B**), and U87 (**C**) tumors in untreated (black dots) and chemo-radiotherapy treated (blue dots) animals at the indicated time points. The 4 day period over which treatment was applied is indicated. Tumor volumes on the day of the first treatment session and at two and seven days post treatment for A11 (**D**), S2 (**E**) and U87 (**F**) tumors. S2 *P < 0.05, **P=0.0035, U87 *P < 0.05.

**
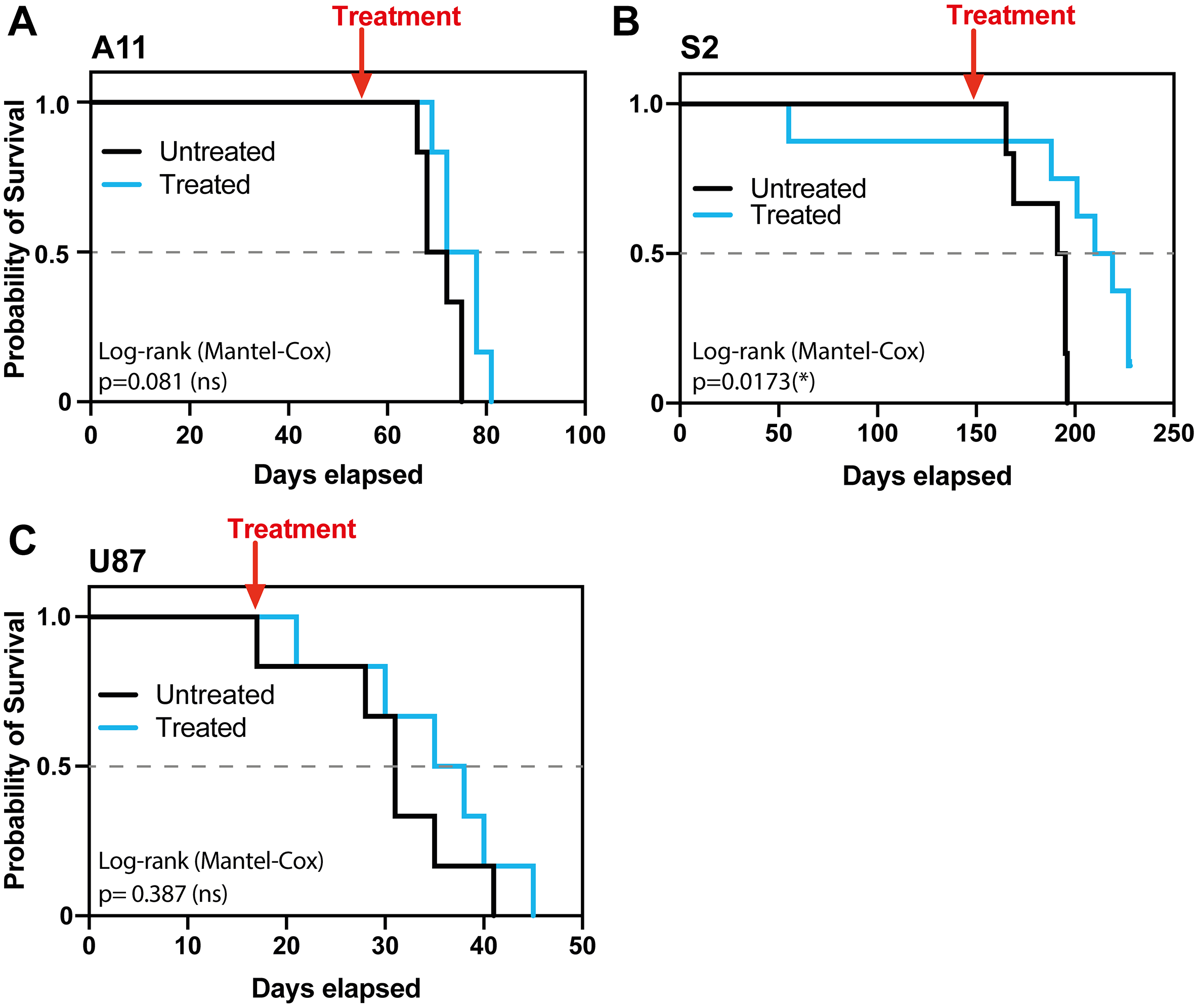
**

**Supplementary Figure 7.** Kaplan-Meier survival curves for tumor-bearing mice that were untreated or treated with chemo-radiotherapy. These data are from the same cohort of animals used for the tumor volume study presented in Supplementary Figure 6. (**A**) A11 tumor-bearing mice. Treatment failed to prolong survival time (n=8 per group, median survival = 70 days for untreated animals vs. 75 days for animals treated with chemo-radiotherapy). (**B**) S2 tumor-bearing mice. Treatment prolonged survival time significantly (n=8 per group, median survival = 195 days for untreated animals vs. 207.5 days for animals treated with chemo-radiotherapy, P=0.017). One of the treated animals was omitted from the analysis as it was found to have hydrocephalus on post mortem investigation. (**C**) U87 tumor-bearing mice, Treatment failed to prolong survival time (n=6 per group, median survival = 31 days for untreated animals vs. 35.5 days for animals treated with chemo-radiotherapy). P values were determined using the log-rank test. Animals were euthanized when they developed neurological symptoms or weight loss that exceeded 15% of initial body weight, which are requirements of our UK Home Office Licence.


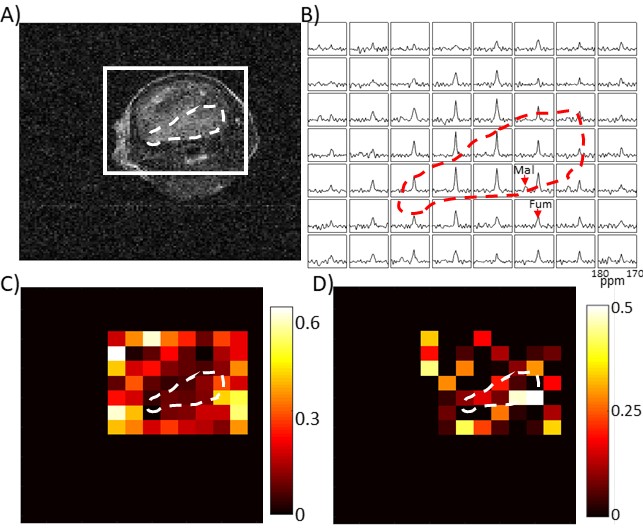


**Supplementary Figure 8**. (**A**) T_2_-weighted ^1^H image of a mouse brain with an S2 tumor. The location of the ^13^C MRSI voxels shown in (**B**) are indicated. The tumor is outlined with a dotted white line. (**B**) ^13^C MRSI spectra from the area shown in (**A**) 20 s after i.v. injection of hyperpolarized [1,4-^13^C, 2,3-^2^H_2_] fumarate. The positions of the large fumarate singlet (Fum) and the two, overlapping, malate resonances (Mal) are indicated. The spectra are displayed with a Lorentzian line broadening of 47 Hz. The position of the tumor, determined from the ^1^H image, is indicated by a dotted red line. Consistent with the spectroscopy data malate signal intensity is very low and observable in only a few voxels. (**C**) Malate/fumarate ratio map for the voxels shown in (**B**). The location of the tumor is outlined. The values in the brain are small (< 0.1) and variable with no discernible difference between tumor and the contralateral brain. (**D**) Map of the malate resonance SNR. This was calculated from the amplitude of the malate resonance and the standard deviation of the noise from an upfield region of the spectrum. The location of the tumor is outlined.
